# Clustering analysis and prognostic model based on PI3K/AKT-related genes in pancreatic cancer

**DOI:** 10.3389/fonc.2023.1112104

**Published:** 2023-04-14

**Authors:** Xiangying Deng, Xu He, Zehua Yang, Jing Huang, Lin Zhao, Min Wen, Xiyuan Hu, Zizheng Zou

**Affiliations:** ^1^ Yiyang Key Laboratory of Chemical Small Molecule Anti-Tumor Targeted Therapy, Department of Biochemistry and Molecular Biology, School of Basic Medical Sciences, Yiyang Medical College, Yiyang, China; ^2^ National Clinical Research Center for Geriatric Disorders, Xiangya Hospital, Central South University, Changsha, China; ^3^ Department of Science and Education, Yiyang Central Hospital, Yiyang, China; ^4^ The Hunan Provincial Key Laboratory of Precision Diagnosis and Treatment for Gastrointestinal Tumor, Xiangya Hospital, Central South University, Changsha, China; ^5^ Department of Pathology, The Second Xiangya Hospital, Central South University, Changsha, China; ^6^ Department of Biochemistry and Molecular Biology, Hunan Province Key Laboratory of Basic and Applied Hematology, Hunan Key Laboratory of Animal Models for Human Diseases, School of Life Sciences, Xiangya School of Medicine, Central South University, Changsha, China

**Keywords:** pancreatic cancer, PI3K/AKT-related genes, bioinformatics, prognostic model, PPP2R3A

## Abstract

**Background:**

Pancreatic cancer is one of most aggressive malignancies with a dismal prognosis. Activation of PI3K/AKT signaling is instrumental in pancreatic cancer tumorigenesis. The aims of this study were to identify the molecular clustering, prognostic value, relationship with tumor immunity and targeting of PI3K/AKT-related genes (PARGs) in pancreatic cancer using bioinformatics.

**Methods:**

The GSEA website was searched for PARGs, and pancreatic cancer-related mRNA data and clinical profiles were obtained through TCGA downloads. Prognosis-related genes were identified by univariate Cox regression analysis, and samples were further clustered by unsupervised methods to identify significant differences in survival, clinical information and immune infiltration between categories. Next, a prognostic model was constructed using Lasso regression analysis. The model was well validated by univariate and multivariate Cox regression analyses, Kaplan−Meier survival analysis and ROC curves, and correlations between risk scores and patient pathological characteristics were identified. Finally, GSEA, drug prediction and immune checkpoint protein analyses were performed.

**Results:**

Pancreatic cancers were divided into Cluster 1 (C1) and Cluster 2 (C1) according to PARG mRNA expression. C1 exhibited longer overall survival (OS) and higher immune scores and CTLA4 expression, whereas C2 exhibited more abundant PD-L1. A 6-PARG-based prognostic model was constructed to divide pancreatic cancer patients into a high-risk score (HRS) group and a low-risk score (LRS) group, where the HRS group exhibited worse OS. The risk score was defined as an independent predictor of OS. The HRS group was significantly associated with pancreatic cancer metastasis, aggregation and immune score. Furthermore, the HRS group exhibited immunosuppression and was sensitive to radiotherapy and guitarbine chemotherapy. Multidrug sensitivity prediction analysis indicated that the HRS group may be sensitive to PI3K/AKT signaling inhibitors (PIK-93, GSK2126458, CAL-101 and rapamycin) and ATP concentration regulators (Thapsigargin). In addition, we confirmed the oncogenic effect of protein phosphatase 2 regulatory subunit B’’ subunit alpha (PPP2R3A) in pancreatic cancer *in vitro* and *in vivo*.

**Conclusions:**

PARGs predict prognosis, tumor immune profile, radiotherapy and chemotherapy drug sensitivity and are potential predictive markers for pancreatic cancer treatment that can help clinicians make decisions and personalize treatment.

## 1 Introduction

Pancreatic cancer is one of the most aggressive solid malignancies and has the highest mortality rate among all primary cancer types (1). Pancreatic cancer is projected to be the second leading cause of cancer deaths by 2030 (2). Pancreatic cancer is notoriously resistant to chemotherapy, targeted therapies, and immunotherapy (3, 4). Despite significant efforts to develop effective treatment strategies, the survival rate for patients with this disease is only 11% at 5 years, with a median survival of less than 11 months (1, 5). The primary reasons for the poor prognosis are the lack of obvious and distinctive symptoms, lack of reliable biomarkers and an incredible capacity for metastasis (6). Therefore, the exploration of new and unique biomarkers is essential for the individualized treatment of tumor patients (7).

The PI3K/AKT pathway participates in both intra- and extracellular signaling and functions in cell survival, growth and proliferation (8–10). The tumorigenic process of pancreatic cancer involves the activation of many signaling pathways, including PI3K/AKT, with an activation rate of approximately 60%, which promotes aggressive tumor behavior and resistance to therapy (11–14). Furthermore, PI3K pathway activity is significantly and negatively correlated with survival in pancreatic cancer patients (15, 16). Therefore, new compounds that are effective in the treatment of pancreatic cancer have been developed based on the PI3K/AKT signaling pathway (14). Furthermore, inhibition of PI3Kδ in T cells increases their sensitivity to pancreatic ductal adenocarcinoma, thereby enhancing their antitumor cell activity (17). However, the exact role of PI3K/AKT in pancreatic cancer patient prognosis, chemotherapy and immunotherapy requires further investigation.

In this study, we first established two clusters that grouped pancreatic cancer cases according to their pathological characteristics and molecular markers based on PARGs. In addition, we constructed a risk model and found that risk scores were significantly associated with tumor metastasis, clustering, immunosuppression, radiotherapy and chemotherapy sensitivity. Finally, we demonstrated that PPP2R3A, which is part of the risk profile, is closely associated with pancreatic cancer metastasis and proliferation.

## 2 Materials and methods

### 2.1 Patients and specimens

On 5 July 2021, mRNA data and clinicopathological profiles of patients with pancreatic cancer were obtained from The Cancer Genome Atlas (TCGA, https://portal.gdc.cancer.gov/). After removal of incomplete information, 182 samples were eventually included in the next data analysis.

The pancreatic cancer tissue samples and clinical information (155 specimens for IHC analysis and 20 specimens for qPCR analysis) originated from the Second Xiangya Hospital of Central South University, and the protocol for the application of the specimens was authorized by the ethics review committee of the institution.

### 2.2 Identification of PARGs

A total of 345 genes in the PI3K/AKT pathway were extracted from the Genome Enrichment Analysis (GSEA, http://www.gsea-msigdb.org/gsea/msigdb/search.jsp). Prognostic and Cox regression analyses using R language yielded 22 PARGs.

### 2.3 Clustering analysis

ConsensusClusterPlus is a tool for unsupervised class discovery. The pancreatic cancer samples were clustered using the ConsensusClusterPlus package (version 1.56.0). The cumulative distribution function (CDF) index reached an approximate maximum when the number of clusters = 2.

### 2.4 Analysis of the degree of immune infiltration in tumor tissue

ESTIMATE (Estimation of STromal and Immune cells in MAlignant Tumor tissues using Expression data) is a package for predicting tumor purity and the presence of stromal/immune cells in tumor tissues using gene expression data. The ESTIMATE (version 2.0.0) package was used to assess the immune score, stromal score and tumor purity score for each pancreatic cancer tissue sample.

### 2.5 Single-sample gene set enrichment analysis (ssGSEA)

Single-sample gene set enrichment analysis (ssGSEA), an extension of the GSEA method, calculates enrichment scores for each sample and gene set pair. Quantification of enrichment scores for immune cells and immune function in pancreatic cancer specimens was performed using the ssGSEA method.

### 2.6 Construction and verification of a PARG-based risk model

The methodology was as described previously (18). Briefly, pancreatic cancer samples from TCGA were randomly divided into training and test groups in a 1:1 ratio using the caret package (version 6.0-91) and further analyzed and validated by Lasso regression analysis and the glmnet R package (version 4.1-3). Lasso is a Cox regression model fit to a single value of lambda by a penalized maximum likelihood method. The model can handle (start, stop) data and stratification as well as sparse design matrices.

The sample was divided into HRS and LRS groups according to the middle of the risk score. Survival and the model were assessed using survival, survminer and timeROC. Kaplan−Meier survival curves and ROC curves for survival were plotted for the training and test groups, respectively. Univariate and multivariate Cox regression analyses confirmed the good independent prognostic value of the model and compared the differences in clinical information across subgroups. Variations in risk scores between subgroups and expression heatmaps for the six PARGs were analyzed with limma (version 3.48.3) and ggpubr (version 0.4.0) software.

### 2.7 Drug sensitivity prediction

pRRophetic uses baseline gene expression levels of cell lines and *in vitro* drug sensitivity to predict clinical drug response. The sensitivity of the HRS and LRS groups to each chemotherapy drug was assessed using pRRophetic (version 2.0.0) software.

### 2.8 Quantitative real-time polymerase chain reaction (qPCR)

RNA samples were extracted from liquid nitrogen-frozen or fresh human pancreatic cancer using TRIzol reagent (15596026, Invitrogen), followed by reverse transcription of mRNA to cDNA using the HiFiScript cDNA Synthesis Kit (R223-1, Vazyme). Finally, real-time quantitative PCR was performed on a 96-well plate on an ABI Prism 7500 system (Applied BioSystems) using the ChamQ Universal SYBR qPCR Master Mix kit (Q711-02, Vazyme). The 2 ^-ΔΔCt^ method was used to calculate the relative expression fold change of each gene, and GAPDH was used as an internal control for the comparison of gene expression data. Primer sequences are presented in **Supplementary Table 1**.

### 2.9 PPP2R3A knockdown

Two PPP2R3A short hairpin RNA (shRNA) lentiviral vectors and a negative control vector were purchased from GenePharma and transfected into HEK293T cells using lipofectamine 3000 to generate and collect lentiviruses, which were subsequently infected with AsPC-1 and Capan-1 cells and screened using 5 µg/mL puromycin.

### 2.10 Western blot

Cells were lysed with RIPA buffer, separated by SDS−PAGE, electrotransferred onto PVDF membranes, blocked with 5% skimmed milk, incubated with the indicated primary and secondary antibodies and finally subjected to chemiluminescence detection.

### 2.11 Cell viability assay

Cells were cultured to logarithmic growth phase, trypsin digested and seeded in 96-well plates at 2000-3000 cells per well for the indicated times. Subsequently, the wells were washed twice with 1× PBS, fixed with 4% PFA for 20 min, dyed with crystal violet 0.1% (C0775, Sigma−Aldrich) for 20 min (20 µl per well), washed with ddH_2_O three times and lysed with 10% acetic acid for 10 minutes. Finally, the absorbance was measured at 590 nm.

### 2.12 Colony formation assays

The procedure was performed essentially as previously described (19). Briefly, cells were treated with trypsin and seeded in 12-well plates (1500 per well with AsPC-1; 500 per well with Capan-1), and the medium was changed every 3 days up to visible colony formation. Subsequently, the cells were washed 2 times with 1 x PBS, fixed in 4% PFA, dyed with 0.1% crystal violet, washed 3 times with 1 x PBS and finally photographed, and the clone number was counted.

### 2.13 Wound healing assay

After trypsin digestion, cells were seeded in 6-well plates (2×10^5^ per well with AsPC-1; 1×10^5^ per well with Capan-1), cultured until the cells formed a confluent monolayer, scribed with a 200 μl pipette tip, rinsed twice with 1× PBS, incubated in medium containing 1% FBS for the indicated time, and photographed under a microscope. The wound width was measured, and the percentage of slit closure was calculated using ImageJ software.

### 2.14 Transwell invasion assay

The procedure was performed essentially as previously described (20). Matrigel was thawed and diluted with serum-free medium (ratio 1:8), followed by the addition of 40 µl of the dilution mixture to the upper chamber (performed on ice). Subsequently, 0.1 ml of 1-1.5 × 10^5^ cell suspension was injected into the upper compartment (1% FBS), and 0.8 ml of culture medium was injected into the lower compartment (10% FBS). After incubation for the indicated time, the chambers were removed, fixed in methanol, stained with 1% crystal violet and photographed with a microscope after the cells were removed from the upper compartment with a cotton swab.

### 2.15 Mouse xenograft tumor models

All animal experiments were performed in accordance with China Food and Drug Administration guidelines. Protocols were reviewed and approved by the Department of Laboratory Animals, Central South University. Four- to six-week-old BALB/c nu/nu female mice were purchased from SJA Laboratory Animal Co., Ltd. A total of 1×10^6^ AsPC-1 cells transfected with PPP2R3A shRNA lentivirus (KD) or empty vector (NC) were injected subcutaneously into the right flank of BALB/c nu/nu female mice. Tumor sizes were measured every three days. Twenty-four days later, the mice were sacrificed, and the tumors were dissected and weighed. Tumor volume was calculated using the equation V (mm^3^) = (a×b^2^)/2, where a is the largest diameter and b is the smallest diameter.

### 2.16 Immunohistochemistry staining (IHC)

The procedure was performed essentially as previously described (21). Briefly, primary antibodies against PPP2R3A (ab218165, Abcam) and Ki-67 (#9449, CST) were diluted 1:250 and subsequently added to tissue sections incubated overnight at 4°C. After three washes in PBS, tissue sections were stained using a nonbiotin horseradish peroxidase detection system (ZSGB-BIO), and hematoxylin-stained nuclei and IHC staining were performed by two independent pathologists for scoring. To evaluate PPP2R3A and Ki-67, a semiquantitative scoring criterion was used in which both the staining intensity and positive areas were recorded. The staining index (values 0-12) was calculated from the product of the positive staining intensity (weak, 1; moderate, 2; strong, 3) and the proportion of positive cells of interest (0%, 0; <25%, 1; 26-50%, 2; 51-75%, 3; >76%, 4).

### 2.17 Statistical analysis

Statistical analyses were performed by R software (version 4.1.3) or GraphPad Prism 8. The chi-squared test was used to analyze the clinicopathological characteristics of patients in the training and test groups, and unpaired two-tailed Student’s *t* tests were used to compare differences between groups. (* p < 0.05; ** p < 0.01; *** p < 0.001).

## 3 Results

### 3.1 Twenty-two PARGs can divide pancreatic cancer into two clusters

**Figure 1:**
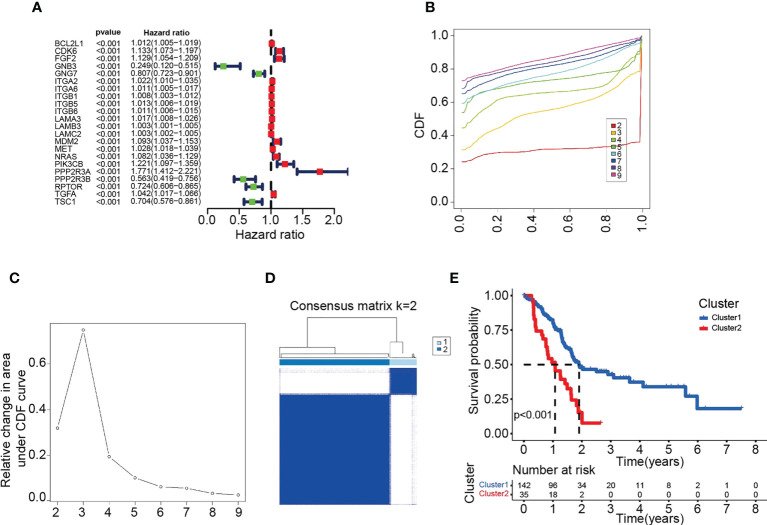
Twenty-two PARG-based scores were used to classify pancreatic cancers into two clusters. **(A)** Forest plot of 22 PARGs based on univariate Cox regression analysis. **(B)** CDF values under different clusters. **(C)** Variation in the area under the CDF curve for different clusters. **(D)** Division of the 182 samples into 2 classes by maximum CDF values. **(E)** OS of patients in different pancreatic cancer clusters. **(A-E)** Data sourced from TCGA.

To investigate the role of PARGs in pancreatic cancer clustering, we performed univariate Cox regression analysis from 346 PARGs and selected 22 PARGs with significant hazard ratios for clustering (**Figure 1A**). The results demonstrated that pancreatic cancer patients were grouped into two clusters, C1 and C2 (**Figures 1B–D**). Subsequently, survival analysis demonstrated a shorter OS for C2 compared with C1 (**Figure 1E**), suggesting that PARGs are closely associated with pancreatic cancer prognosis.

### 3.2 Clustering associated with immune checkpoints and tumor infiltrating lymphocytes (TIL)

**Figure 2:**
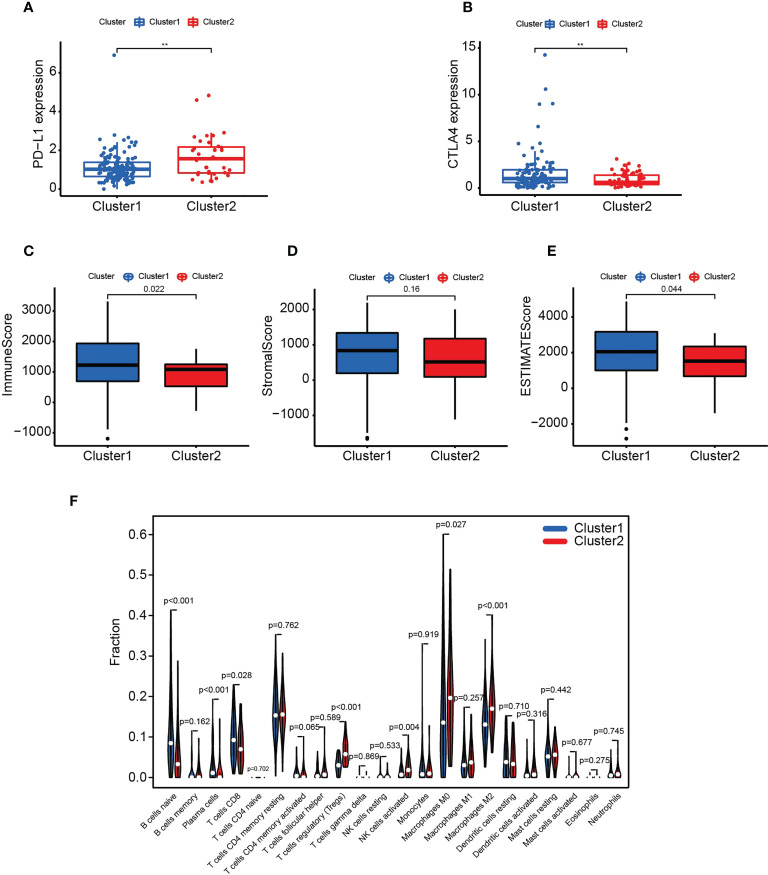
Clustering associated with immune checkpoints and TILs. **(A-F)** Comparison of PD-L1 **(A)**, CTLA-4 **(B)**, immune score **(C)**, stromal score **(D)**, ESTIMATE score **(E)** and TILs **(F)** in C1 and C2. ** p < 0.01. **(A-F)** Data sourced from TCGA.

We further investigated the relationship between clustering and PD-L1 or CTLA-4. The two immune checkpoint proteins were differentially expressed in C1 and C2 (**Figures 2A, B**), with PD-L1 highly expressed in C2 and CTLA-4 highly expressed in C1.

Immune and ESTIMATE scores were higher in C1 than in C2 (**Figures 2C–E**). Furthermore, antitumor immune cell populations such as naive B cells, plasma cells and CD8 T cells were more abundant in C1. In contrast, activated NK cells, regulatory T cells (Tregs), and M0 and M2 macrophages were higher in C2 (**Figure 2F**).

### 3.3 Construction and verification of the six PARG-based risk model

**Figure 3:**
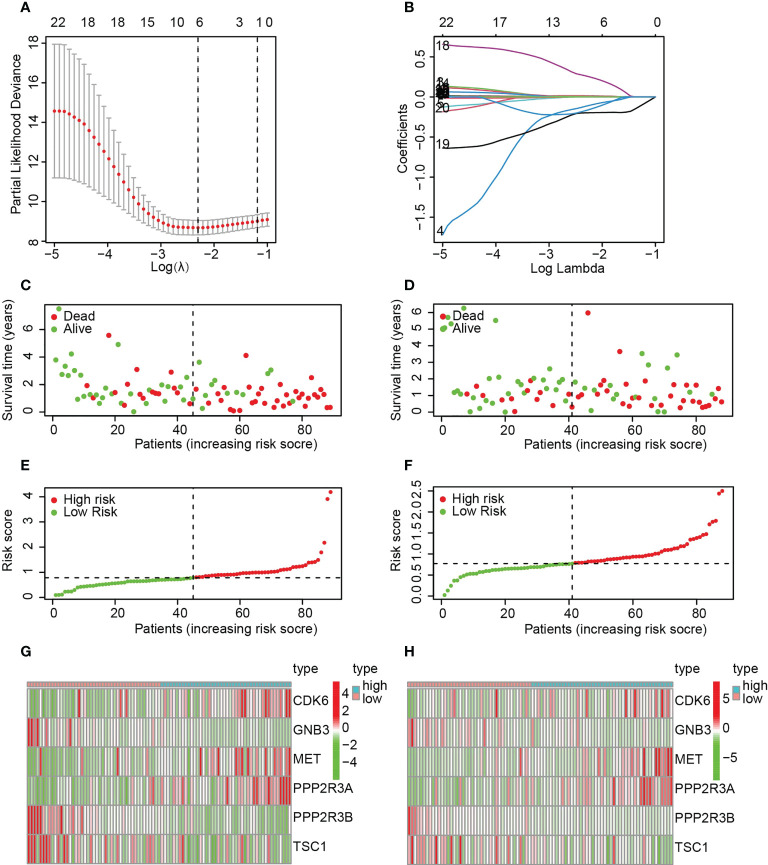
The construction of a risk model based on six PARGs. **(A, B)** Identification of six prognosis-related PARGs. **(C, D)** Survival status of pancreatic cancer patients in the training **(C)** and test **(D)** groups. **(E, F)** Risk scores of pancreatic cancer patients in the training **(C)** and test **(D)** groups. **(G, H)** The expression of six prognosis-related PARGs in the HRS and LRS groups in the training **(G)** and test groups **(H)**. **(A-H)** Data sourced from TCGA.

To construct a PARG-based risk model, we performed Lasso regression analysis on 22 PARGs and selected CDK6, GNB3, MET, PPP2R3A, PPP2R1B and TSC1 to construct a prediction model (**Figures 3A, B**). Patients were classified into training and test groups, and analysis of the clinicopathological characteristics demonstrated no significant differences between them (**Table 1**). **Figure 3** presents the risk scores, survival status and expression of the six PARGs in the training group (**Figures 3C, E, G**) and the test group (**Figures 3D, F, H**).

**Table 1:** Clinical features of the training and testing sets.

Variables	Train sets (89)		Test sets (88)		p value
	NO.	%	NO.	%	·
Age					
** <=60**	31	34.83	27	30.68	
** >60**	58	65.17	61	69.32	0.6316
Gender					
** Female**	46	51.69	34	38.64	
** Male**	43	48.31	54	61.36	0.0971
Stage					
** I**	13	14.61	8	9.09	
** II**	72	80.90	74	84.09	
** III**	1	1.12	2	2.27	
** IV**	2	2.25	2	2.27	
**Unknown**	1	1.12	2	2.27	0.6757
T					
** 1**	5	5.62	2	2.27	
** 2**	16	17.98	8	9.09	
** 3**	66	74.16	75	85.23	
** 4**	1	1.12	2	2.27	
**Unknown**	1	1.12	1	1.14	0.1828
N					
** 0**	25	28.09	24	27.27	
** 1**	63	70.79	60	68.18	
**Unknown**	1	1.12	4	4.55	0.9812
M					
** 0**	42	47.19	37	42.05	
** 1**	2	2.25	2	2.27	
**Unknown**	45	50.56	49	55.68	0.9015
Grade					
** 1**	19	21.35	12	13.64	
** 2**	45	50.56	49	55.68	
** 3**	22	24.72	26	29.55	
** 4**	2	2.25	0	0.00	
**Unknown**	1	1.12	1	1.14	0.2531

**Figure 4:**
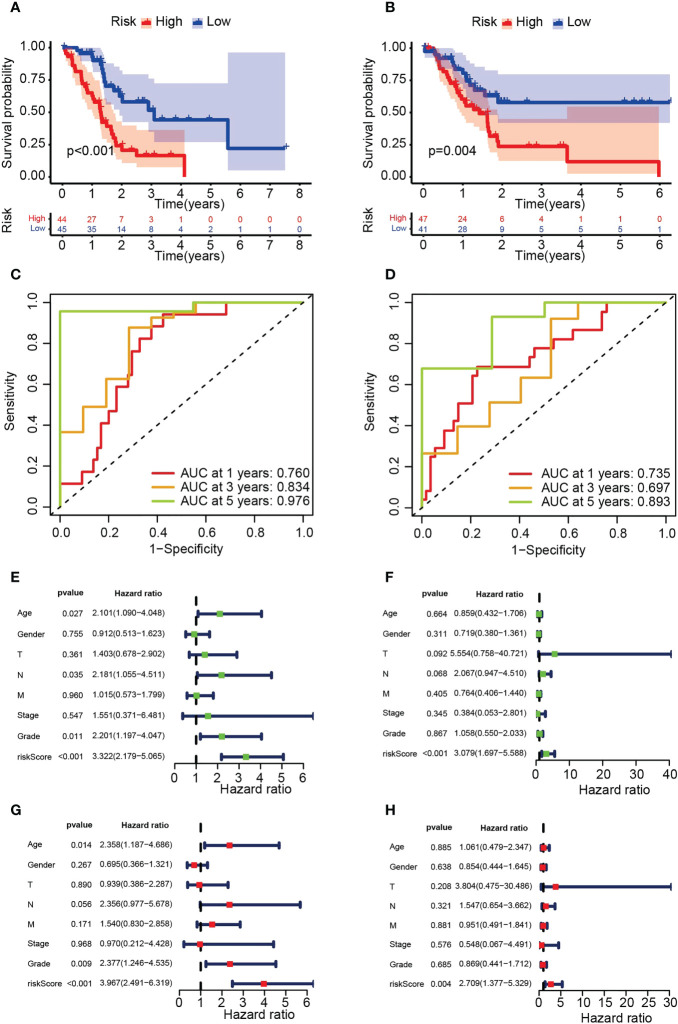
Verification of the risk model based on six PARGs. **(A, B)** OS of the HRS and LRS groups in the training **(A)** and testing **(B)** groups. **(C, D)** AUC for 1-, 3- and 5-year OS in the training **(C)** and test **(D)** groups. **(E-H)** Univariate **(E, F)** or multivariate **(G, H)** Cox regression analysis was used to analyze the risk scores and clinicopathological characteristics of the training **(E, G)** and testing **(F, H)** groups. **(A-H)** Data sourced from TCGA.

**Figure 5:**
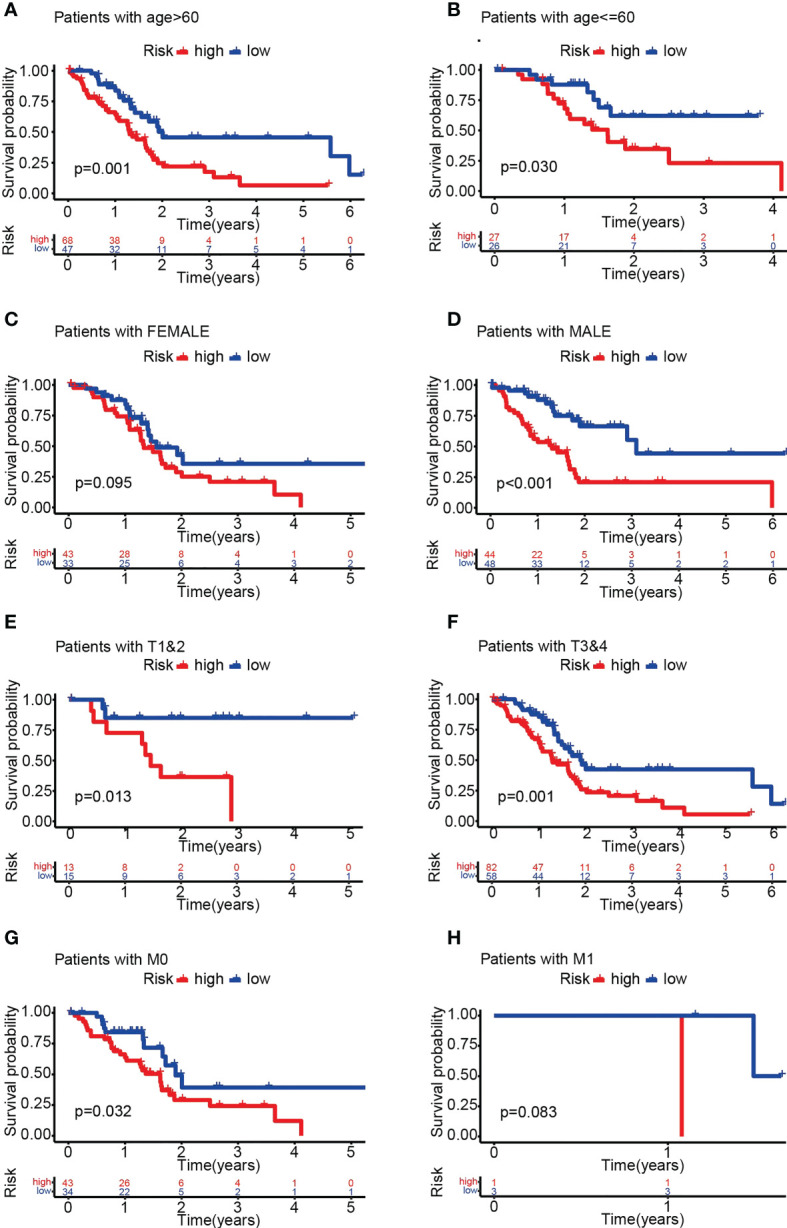
OS analysis of different subgroups of pancreatic cancer in the HRS and LRS groups. **(A-H)** OS of the HRS and LRS groups in subgroups >60 years old **(A)**, ≤60 years old **(B)**, female **(C)**, male **(D)**, T1-2 **(E)**, T3-4 **(F)**, M0 **(G)** and M1 **(H)**. **(A-H)** Data sourced from TCGA.

Survival analysis demonstrated significantly lower OS in the HRS group (**Figures 4A, B**). In addition, the area under the ROC curve (AUC) data for 1-year, 3-year and 5-year OS were 0.760, 0.834, and 0.976 (training group) and 0.735, 0.697, and 0.893 (test group), respectively (**Figures 4C, D**). Risk scores were significant predictors of poorer survival relative to age, sex and stage, suggesting that the model based on PARG risk scores may be more valid than pathological agents for predicting patient survival (**Figures 4E–H**). We also analyzed OS in the HRS and LRS groups classified into age, sex and tumor stage subgroups and found that OS was shorter in the HRS group regardless of age, sex (male) and tumor stage (T1-2, T3-4, M0, N0, N1, Stage I-II, G1-2 and G3-4) (**Figures 5A–H**, **S1A-F**). In conclusion, PARG-based risk models can be applied to older and younger patients, to women and men, and to patients with early and advanced disease.

### 3.4 Six PARG-based risk score correlates with tumor immune status and metastasis

**Figure 6:**
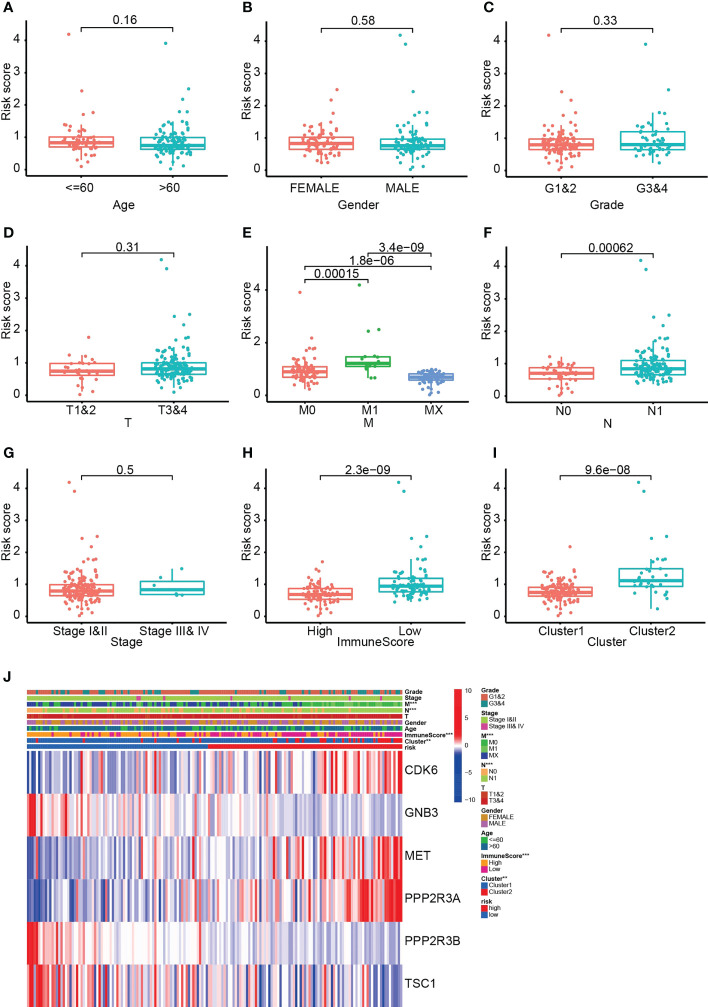
The six PARG-based risk scores are correlated with metastases, immune scores and clusters of pancreatic cancer. **(A-I)** Comparative analysis of risk scores in **(A)** different age groups, **(B)** different sex groups, **(C)** G1-1 and G3-4, **(D)** T1-2 and T3-4, **(E)** M0, M1 and Mx, **(F)** N0 and N1, **(G)** stages I-II and III-IV, **(H)** ImmuneScore groups and **(I)** clusters of patients. **(J)** Heatmap of the relationship between the six PARGs and clinicopathological characteristics. **p < 0.01. ***p < 0.001. **(A-J)** Data sourced from TCGA.

Comparison of risk scores between different subgroups demonstrated significant differences between M0 and M1, N0 and N1, C1 and C2, and high and low immune scores. Patients with C1, no metastases or high immune scores had lower risk scores and a better prognosis (**Figures 6A–I**). In addition, CDK6, MET and PPP2R3A were highly expressed in the HRS group, whereas GNB3, PPP2R3B and TSC1 exhibited the opposite trend (**Figure 6J**). These discoveries suggest that PARGs are correlated with immunological status and distant metastasis in pancreatic cancer.

### 3.5 The HRS group is strongly associated with pancreatic cancer immunosuppression

**Figure 7:**
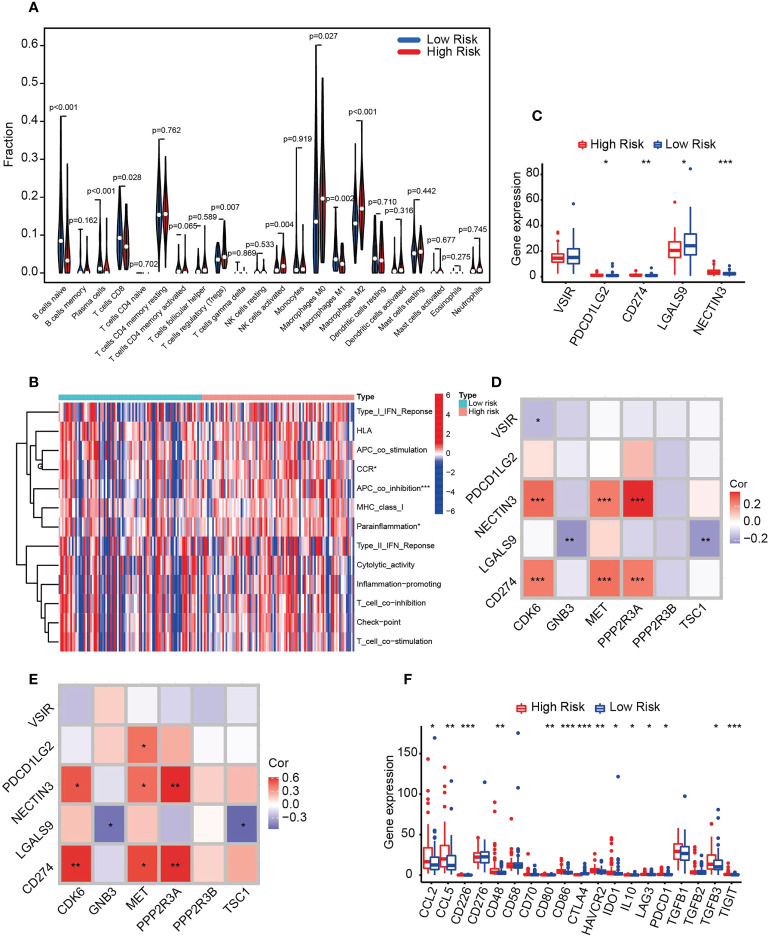
HRS predicts immunosuppression in pancreatic cancer. **(A)** Violin plot of immune cell infiltration in the HRS and LRS groups. **(B)** Heatmap showing the relationships between the risk score and immune functions. **(C)** Box plot of the distribution of immune checkpoint molecules associated with APC_co_inhibition between the HRS and LRS groups. *p < 0.05. **p < 0.01. ***p < 0.001. **(D)** Correlation of six prognosis-related PARGs with five APC_co_inhibition-related immune checkpoints. *p < 0.05. **p < 0.01.***p < 0.001. **(E)** qPCR was used to detect the expression of the molecules indicated in the figure in human pancreatic cancer specimens and to perform correlation analysis. n = 20, *p < 0.05. **p < 0.01. ***p < 0.001. **(F)** Box plots comparing the expression of immunosuppressive biomarkers between the HRS and LRS groups. *p < 0.05. **p < 0.01. ***p < 0.001. **(A-D, F)** Data sourced from TCGA.

To assess the association of risk scores and pancreatic cancer immunity, we calculated the abundance of 22 immune-related cell types and demonstrated that the predominant tumor-infiltrating immune cells in the HRS group were Tregs, activated NK cells and M0 and M2 macrophages (**Figure 7A**). In contrast, naive B cells, plasma cells, activated CD8 T cells and M1 macrophages were more abundant in the LRS group. Therefore, these results suggest a potential association between HRS and immunosuppression.

We further tested the transcript expression of immunosuppressive molecules between the HRS and LRS groups and demonstrated that APC_co_inhibition, CCR and parinflamation were markedly different between them, with APC_co_inhibition being the most pronounced (**Figure 7B**). We then further analyzed the immune molecules included in this group. NECTIN3, CD274 and PDCD1LG2 were hyperexpressed in the HRS group, whereas LAGLS9 was low (**Figure 7C**), indicating that inhibitors targeting NECTIN2, CD272 and PDCD1LG2 were more effective in the HRS group. Moreover, we analyzed the correlation between APC_co_inhibition component molecules and six PARGs and determined that CD274 and NECTIN3 were positively correlated with the HRS molecules CDK6, MET and PPP2R3A, whereas LGALS9 was negatively correlated with GNB3 and TSC1 (**Figure 7D**). To verify the accuracy of the results, we collected specimens from 20 clinical pancreatic cancer patients for qPCR validation, and the results were generally consistent with the above findings (**Figure 7E**), indicating that the data were reliable. Finally, we compared the transcript expression of immunosuppressive biomarkers, including immune checkpoint markers and secreted immunosuppressive factors, between the HRS and LRS groups. The results of the correlation analysis demonstrated that CCL2, CCL4, CD226, CD48 CD80, CD86, HAVCR2, IDO1, IL10, LAG3, PDCD1 (PD-1), TGFB3 and TIGIT were positively correlated with HRS, with the exception of CTLA4, which was negatively correlated (**Figure 7F**). Taken together, the above results suggest that six PARGs are instrumental in regulating the immune status of tumors.

### 3.6 HRS correlates strongly with treatment sensitivity in pancreatic cancer

**Figure 8:**
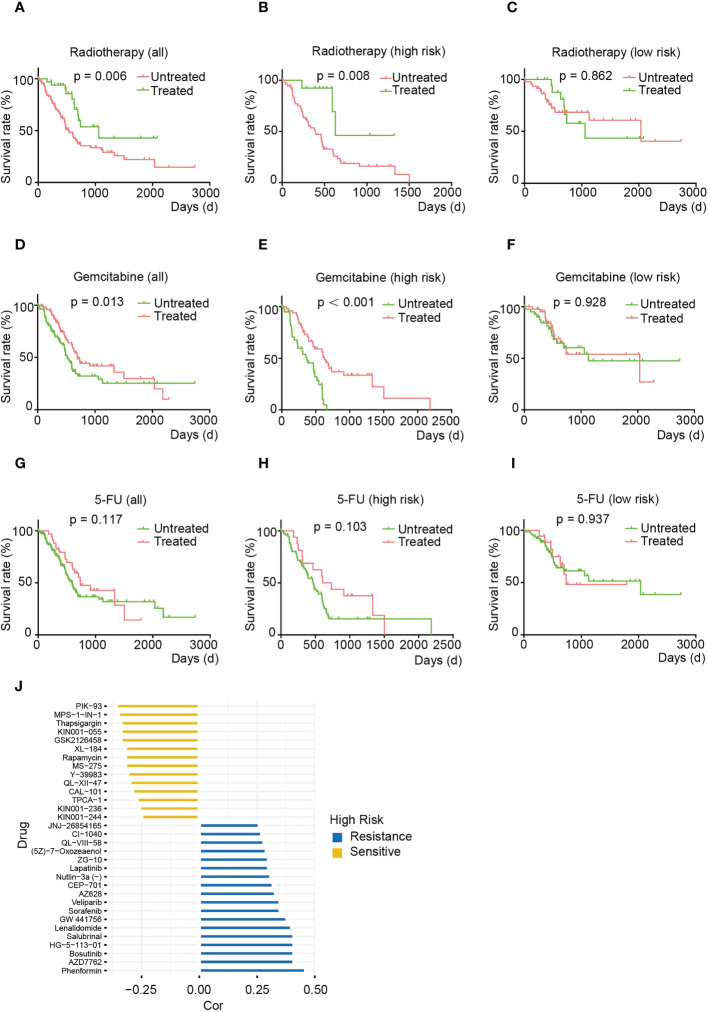
The HRS is strongly associated with treatment sensitivity phenotypes in pancreatic cancer. **(A-C)** OS of total **(A)**, HRS **(B)** and LRS **(C)** pancreatic cancer patients treated with or without irradiation. **(D-F)** OS of total **(D)**, HRS **(E)**, and LRS **(F)** pancreatic cancer patients treated with or without gemcitabine. **(G-I)** OS of total **(G)**, HRS **(E)**, and LRS **(F)** pancreatic cancer patients after treatment with or without 5-FU. **(J)** Multidrug sensitivity analysis for patients in the HRS group. **(A-J)** Data sourced from TCGA.

Radiotherapy and chemotherapy are the first-line treatments in the clinical management of pancreatic cancer (22, 23). To further investigate whether risk features correlate with treatment response, we compared OS in patients treated with radiotherapy, gemcitabine or 5-FU and untreated patients in the HRS and LRS groups. Specifically, the relationship between radiation therapy (**Figures 8A–C**) or guitarcitabine chemotherapy (**Figures 8D–F**) and risk score exhibited a similar pattern, with both treatments improving the prognosis of patients with HRS, but there was no significant difference between the treated and nontreated groups for patients with LRS. For 5-FU, there was no significant improvement in OS with this therapy in either the HRS or LRS group (**Figures 8G–I**). The above results demonstrate that the HRS may be a valid marker for pancreatic cancer patients undergoing radiotherapy and gemcitabine treatment. However, these results are inconclusive for 5-FU treatment.

To gain more insight into the association between risk score and drug response, we analyzed sensitivity to therapeutic agents using the pRRophetic package. We found that the HRS group may be resistant to AMPK activators (phenylephrine), Chk inhibitors (AZD7762) and Src inhibitors (bostatinib) that regulate cell growth and differentiation but may possibly be sensitive to PI3K/AKT/mTOR signaling pathway inhibitors (e.g., PIK-93, GSK2126458, CAL-101 and rapamycin) and ATP synthase inhibitors (thapsigargin) (**Figure 8J**). Together, these results suggest that the PARG-based risk model can be used to develop novel treatments for pancreatic cancer.

### 3.7 PPP2R3A functions as a tumor-promoting factor in pancreatic cancer

**Figure 9:**
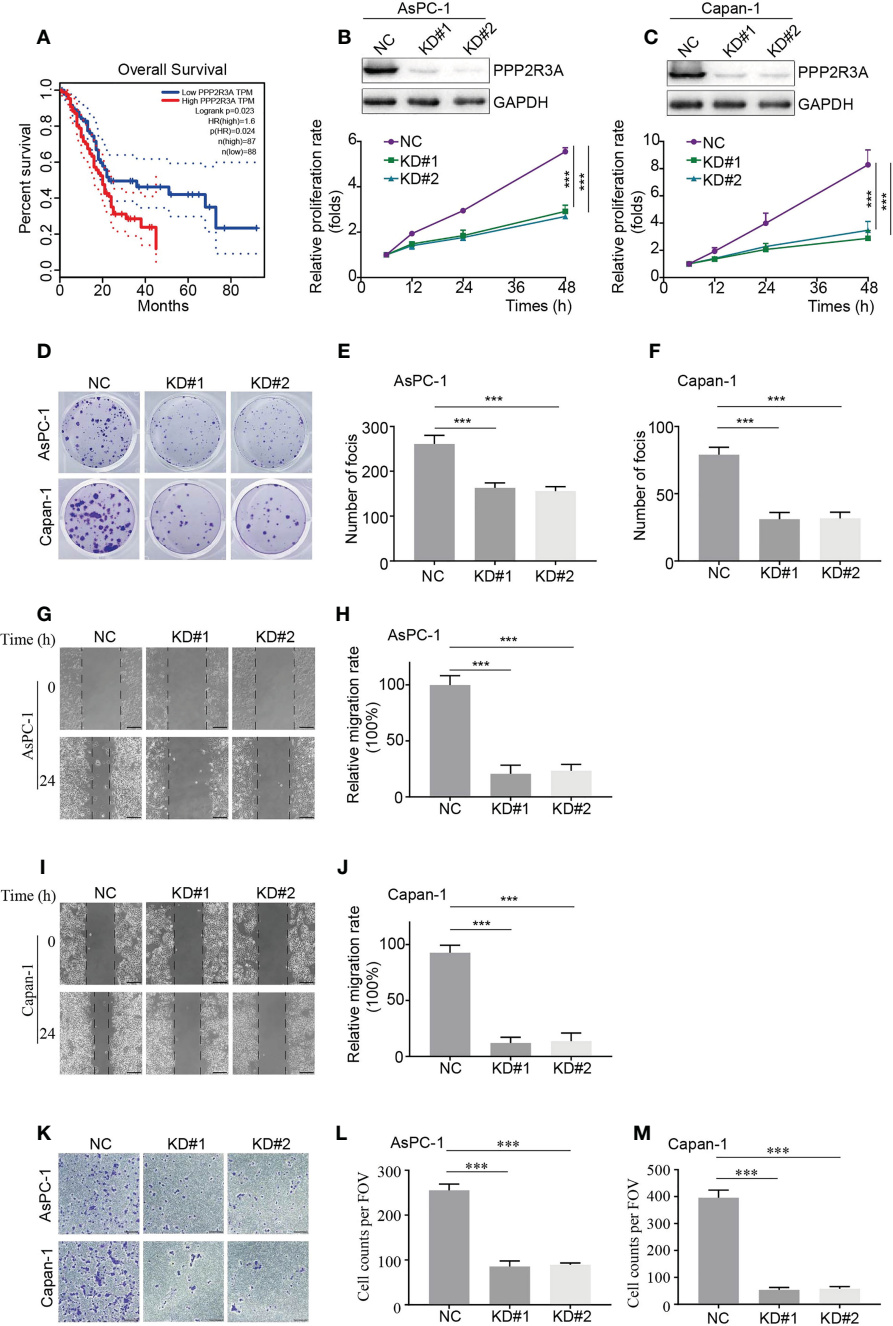
PPP2R3A functions as a tumor-promoting factor in pancreatic cancer (*in vitro* assays). **(A)** OS analysis based on PPP2R3A expression. Data sourced from TCGA. **(B, C)** Western blot assay for expression of the described proteins in AsPC-1 (**B**, top) and Capan-1 (**C**, top) cells transfected with PPP2R3A shRNA lentivirus or empty vector, followed by cell viability assay for proliferation rate (**B, C**, bottom) (mean ± s.d., n = 3). *** p < 0.001. **(D-F)** Colony formation of AsPC-1 (d, top) and Capan-1 (d, bottom) cells transfected with PPP2R3A shRNA lentivirus or empty vector. The total number of foci per well was counted **(E, F)** (mean ± s.d., n = 3). ***p < 0.001. **(G-J)** Wound healing assay of AsPC-1 **(G)** and Capan-1 **(I)** cells transfected with PPP2R3A shRNA lentivirus or empty vector. The distances were measured by ImageJ software **(H, J)** (mean ± s.d., n = 3). ***p < 0.001. Scale bars are 200 μm. **(K-M)** Transwell invasion assay of AsPC-1 (**K**, top) and Capan-1 (**K**, bottom) cells transfected with PPP2R3A shRNA lentivirus or empty vector. The total number of cells per field was counted **(L, M)** (mean ± s.d., n = 3). ***p < 0.001. Scale bars are 200 μm.

PPP2R3A belongs to the PP2A regulatory subunit B” family, and its role in pancreatic cancer tumorigenesis is rarely reported (24). Our results demonstrate that PPP2R3A is highly expressed in the HRS group and in C1 (**Figures 3G, H**, **6J**), suggesting that this protein may be a tumor-promoting factor. To verify this hypothesis, we used Kaplan−Meier analysis to explore the correlation between PPP2R3A expression and OS; patients with high PPP2R3A expression had a shorter OS (**Figure 9A**).

**Figure 10:**
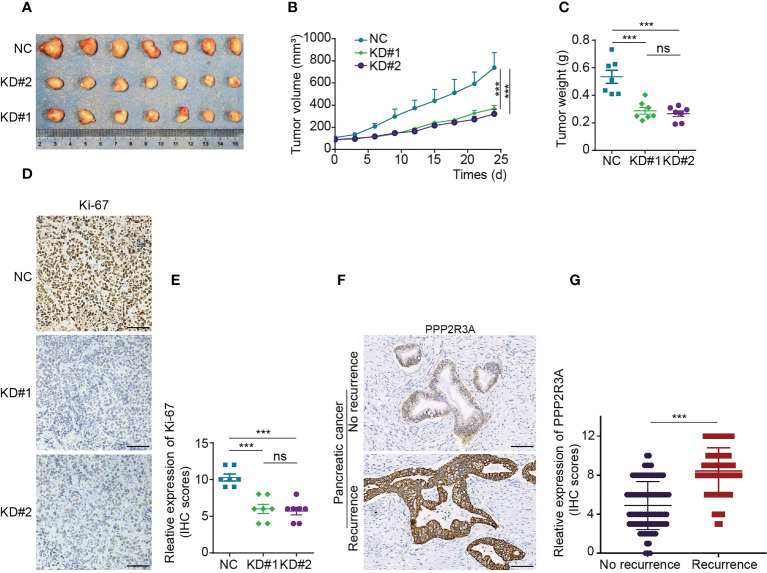
PPP2R3A functions as a tumor-promoting factor in pancreatic cancer (*in vivo* experiments and clinical information). **(A-C)** A total of 1×10^6^ AsPC-1 cells transfected with PPP2R3A shRNA lentivirus (KD) or empty vector (NC) were injected subcutaneously into the right flank of BALB/c nu/nu female mice. Tumor sizes were measured every three days **(B)**. Twenty-four days later, the mice were sacrificed, and the tumors were dissected and weighed **(A, C)**. (mean ± s.d., n = 7). ***p < 0.001, ns: not significant (p > 0.05). **(D)** IHC staining map of Ki-67 in tumor tissues. Scale bar is 100 μm. **(E)** Ki-67 expression score in tumor tissues. ***p < 0.001, ns, not significant (p > 0.05). **(F)** IHC staining map of PPP2R3A in human pancreatic cancer tissue. Scale bar is 100 μm. **(G)** PPP2R3A expression scores in recurrent (n=65) and recurrence-free (n=90) human pancreatic cancer. ***p < 0.001.

To further investigate the role of PPP2R3A, we selected two pancreatic cancer cell lines with high metastatic activity, AsPC-1 and Capan-1, and established stable PPP2R3A knockdown cell lines by transfection with PPP2R3A shRNA lentivirus. We then detected the relative proliferation rate and clone formation ability of the cells. After knockdown of PPP2R3A (**Figures 9B, C**, top), the proliferation rate of cells decreased (**Figures 9B, C**, bottom), the clonal sphere size decreased, and the number of clones decreased (**Figures 9D–F**). Importantly, wound healing assays and transwell results demonstrated that tumor cell migration (**Figures 9G–J**) and invasion (**Figures 9K–M**) were also significantly reduced after PPP2R3A knockdown. Furthermore, knockdown of PPP2R3A in AsPC-1 cells significantly inhibited xenograft tumor growth in mouse models (**Figures 10A–E**). In addition, we analyzed the expression of PPP2R3A in clinical human pancreatic cancer tissue samples (**Figure 10F**) and found that upregulation of PPP2R3A expression was significantly associated with tumor recurrence (**Figure 10G**). Overall, these results suggest that PPP2R3A is a tumor-promoting factor in pancreatic cancer.

## 4 Discussion

Treating pancreatic cancer is a serious clinical problem because of it typically presents in advanced stages and has a poor prognosis (25). Although molecular subtyping now guides drug development and clinical treatment for many cancer types, pancreatic cancer subtyping does not currently influence therapeutic decisions. However, molecular identification of diagnostic biomarkers and therapeutic targets for pancreatic cancer should be prioritized (7). PI3K/AKT signaling is activated in 60% of pancreatic cancer patients and plays an important role in pancreatic cancer tumorigenesis (12, 14, 16). Therefore, the purpose of this study was to construct PARG-related clustering and risk models to provide a reference for the clinical treatment of pancreatic cancer.

We screened six PARGs to construct a prognostic model. Of these six genes, three were significantly associated with poor prognosis, including CDK6, MET and PPP2R3A. For example, evidence suggests that overexpression of D-type cyclins or inactivation of the INK4 protein family results in CDK6 often remaining activated in tumors (26). Downregulation of CDK6 leads to inhibition of pancreatic cancer cell metastatic capacity, whereas increased CDK6 expression was observed in pancreatic cancer clinical samples and was associated with poor prognosis (27). MET was observed to be upregulated in pancreatic cancer, associated with tumor grade, and positively correlated with PD-L1 levels (28, 29). GNB3, PPP2R3B and TSC1 were highly expressed in the LRS group, suggesting that these genes may be tumor suppressors of pancreatic cancer. Recent studies have reported that in a number of human cancers, including pancreatic cancer, TSC1 exhibits tumor suppressive effects (30).

The PPP2R3A gene encodes two proteins, PR130 and PR72, which are regulatory subunits of protein phosphatase 2A (PP2A) (24). PR72 expression is restricted to heart and skeletal muscle tissues, and PR130 is present in multiple tissues (31, 32). PP2A is a serine/threonine phosphatase that can act as a promoter or inhibitor of tumors and is involved in a variety of intracellular signaling pathways, including PI3K/AKT, MYC, WNT/β-catenin, and EGF/EFGR signaling (24, 33–35), where PP2A can directly dephosphorylate AKT, MYC and β-catenin, thereby regulating signaling pathway activity (36–43).I In hepatocellular carcinoma cells, a decrease in PPP2R3A resulted in G1/G2 arrest, a decrease in Ki67, and an increase in P53 and P21 (44). Further studies have found that PPP2R3A gene overexpression promotes HK1 levels and glycolysis, thereby promoting the proliferation, invasion and migration of hepatocellular carcinoma (45). Additionally, PPP2R3 expression is associated with poor prognosis after liver transplantation in hepatocellular carcinoma patients (46). However, the role of PPP2R3A in pancreatic cancer is unclear. Therefore, we validated the role of PPP2R3A in pancreatic cancer and determined that high expression of PPP2R3A was significantly and positively associated with pancreatic cancer proliferation, migration, invasion and poor prognosis. Next, we aim to investigate the molecular mechanisms of PPP2R3A in pancreatic cancer tumorigenesis.

We observed lower risk scores in stage M0 than in stage M1 and in stage N0 than in stage N1, suggesting that PI3K/AKT signaling is a risk factor for distant pancreatic cancer metastasis, which is consistent with the results that high expression of CDK6 (27), MET (28, 29) and PPP2R3A (**Figures 9G–M**) promotes tumor metastasis. However, risk scores for older patients or patients with higher tumor stage did not match prognosis, suggesting that risk scores to predict prognosis in pancreatic cancer patients need to be combined with clinicopathological features.

Studies have demonstrated that pancreatic cancer exhibits significant immunosuppression and is not susceptible to immunotherapy (6, 47, 48). To analyze the causes and identify new potential immunotherapeutic targets, we analyzed the relationship between risk scores and tumor microenvironment and found that activated CD8 T cells and M1 macrophage cell aggregation were increased in the LRS group, whereas both M2 macrophage and Treg cell abundance was higher in the HRS group. Infiltration of CD8+ T cells, CD4+ T cells, NK cells and M1 macrophages kill tumor cells, whereas Treg and M2 macrophages promote tumor growth and proliferation (49–51). NK cells, although more abundant in the HRS group, and their antitumor function may be inhibited. For example, both activated pancreatic stellate cells and tumor-derived extracellular vesicles can inhibit the function of NK cells in the human pancreatic cancer microenvironment (52, 53). Next, the association between the risk model and immune function and immunosuppressive biomarkers was investigated. In addition to CTLA4, which was highly expressed in the LRS group, APC_co_repression, CD274 (PD-L1), HAVCR2, IDO1, LAG3, PDCD1 (PD-1), TGFB3 and TIGIT were highly expressed in the HRS group, suggesting that the HRS group exhibited a significant immunosuppressive state and that the risk model could be used as a new marker for immunotherapy in pancreatic cancer.

Our study determined that risk characteristics predicted clinical response to treatment and that patients with HRS were likely to achieve better outcomes with radiotherapy and gemcitabine chemotherapy. Furthermore, we found that pancreatic cancer cell lines with HRS may be sensitive to multiple antitumor inhibitors, primarily focusing on PI3K/AKT/mTOR signaling inhibitors (PIK-93, GSK2126458, CAL-101 and rapamycin) and modulators of ATP levels (AZD7762 and thapsigargin), consistent with the finding that PI3K/AKT is highly expressed in pancreatic cancer and promotes tumor metastasis. Therefore, these findings suggest the utility of our risk profile in assessing treatment response, especially in HRS patients. However, detailed functional analyses are required to validate our findings.

In summary, we have characterized and defined a new PARG-based clustering and risk model that could serve as a promising predictive device in the clinical management of pancreatic cancer.

## Data availability statement

Publicly available datasets were analyzed in this study. The mRNA expression data and matching clinical information used in this study were obtained from the TCGA database (https://portal.gdc.cancer.gov/projects/TCGA-PAAD).

## Ethics statement

The studies involving human participants were reviewed and approved by the ethical review committee of the Second Xiangya Hospital of Central South University. The patients/participants provided their written informed consent to participate in this study. The animal study was reviewed and approved by the ethical review committee of the Second Xiangya Hospital of Central South University.

## Author contributions

XD performed data analysis and wrote most of the manuscript. XD, JH, MW, and XYH performed the experiments, and XH, ZY, and LZ collected the data and conducted the literature search. ZZ initiated the project, designed the experiments, and wrote and revised the manuscript. All authors contributed to the article and approved the submitted version.
