# Supplementary material for: Clustering analysis and prognostic model based on PI3K/AKT-related genes in pancreatic cancer
Source: Front Oncol. 2023 Apr 14;13:1112104. doi: 10.3389/fonc.2023.1112104 (PMC10140326; doi:10.3389/fonc.2023.1112104)
Supplement: Supplementary file 1 [file DataSheet_1.pdf]

Supplementary Material

1 Supplementary Figures

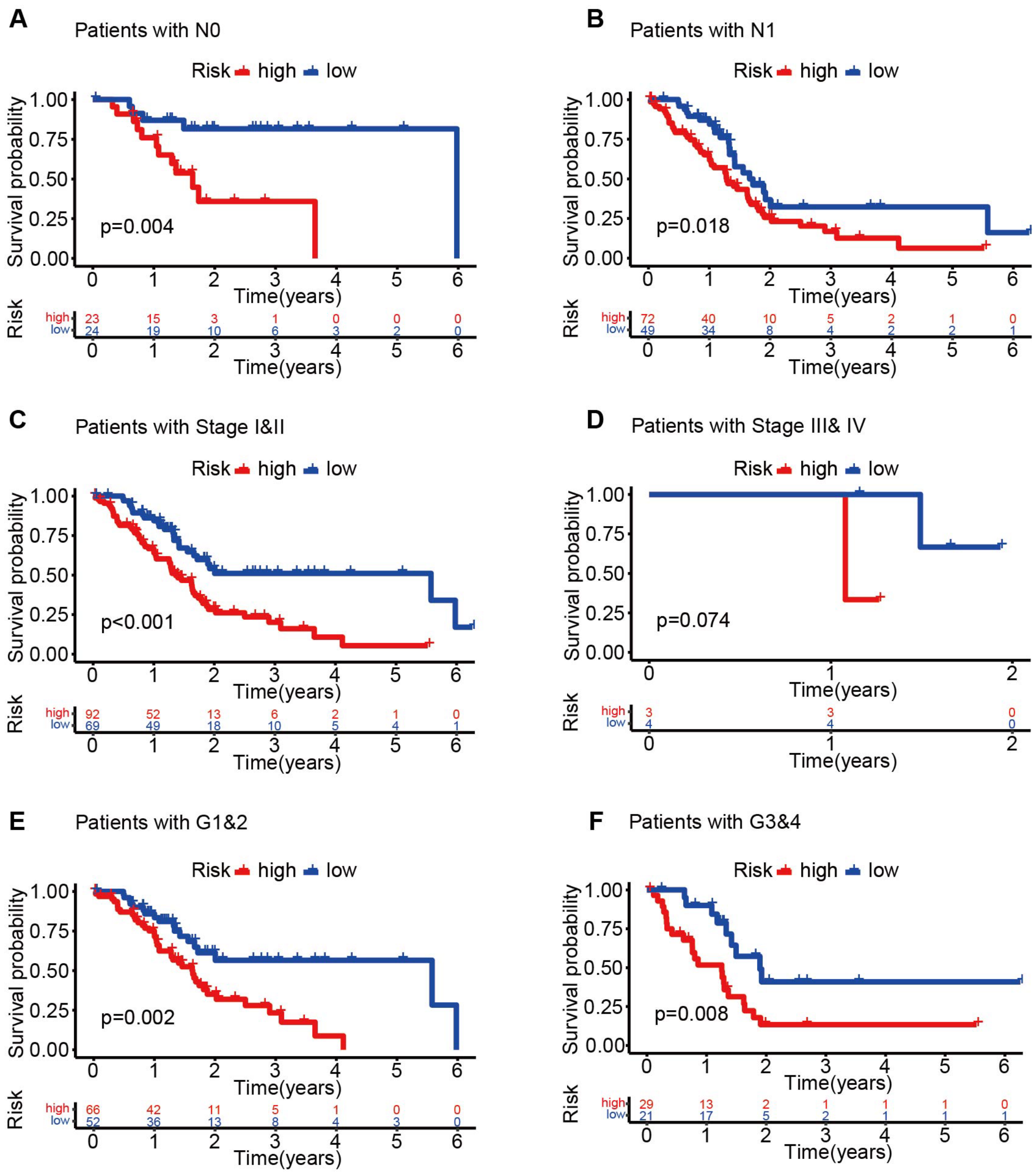

Supplementary Figure 1. OS analysis of different subgroups of PC in HRS and LRS groups. (A-F) OS of HRS and LRS groups in subgroups N0 (A) , N1 (B) , Stage I-II (C) , Stage III-IV (D) , G1-2 (E) and G3-4 (F). (A-F) Data sourced from TCGA.

2 Supplementary Table

Supplementary Table 1. Primers list.

Gene name	Assay	Sense	Sequence (5'-3')
PPP2R3A	qRT-PCR	Forward	CTGTCCTCTCTATTGGAAAGCCC
		Reverse	TGAATGACTGTGCTGTCACAAAT
VSIR	qRT-PCR	Forward	ACGCCGTATTCCCTGTATGTC
		Reverse	TTGTAGAAGGTCACATCGTGC
PDCD1LG2	qRT-PCR	Forward	ATTGCAGCTTCACCAGATAGC
		Reverse	AAAGTTGCATTCCAGGGTCAC
NECTIN3	qRT-PCR	Forward	GCAGTTCACCATCCCCAATATG
		Reverse	TCCAAGCGGGAATGTAACAGC
LGALS9	qRT-PCR	Forward	TCTGGGACTATTCAAGGAGGTC
		Reverse	CCACTGGAGCTGAGAACGG
CD274	qRT-PCR	Forward	TGGCATTTGCTGAACGCATTT
		Reverse	TGCAGCCAGGTCTAATTGTTTT
CDK6	qRT-PCR	Forward	GCTGACCAGCAGTACGAATG
		Reverse	GCACACATCAAACAACCTGACC
GNB3	qRT-PCR	Forward	CGGACGTTAAGGGGACACC
		Reverse	CGAGGCACTTACCAGCAGC
MET	qRT-PCR	Forward	AGCAATGGGGAGTGTAAAGAGG
		Reverse	CCCAGTCTTGTACTCAGCAAC
PPP2R3B	qRT-PCR	Forward	TCCGCAGGGACGAGAGTAG
		Reverse	AAGGTCGGAATGCTTTGGCTC
TSC1	qRT-PCR	Forward	CAACAAGCAAATGTCGGGGAG
		Reverse	CATAGGGCCACGGTCAGAA
GAPDH	qRT-PCR	Forward	GGAGCGAGATCCCTCCAAAAT
		Reverse	GGCTGTTGTCATACTTCTCATGG
